# Association Between the Volume Transfer Constant (K^trans^) From Dynamic Contrast‐Enhanced Perfusion MR and HER2 Status in Breast Cancer Brain Metastases

**DOI:** 10.1002/cnr2.70354

**Published:** 2025-10-01

**Authors:** Jonathan R. Young, Luke N. Ledbetter, Julie A. Ressler, Mark S. Shiroishi, Joanne E. Mortimer, Daniel Schmolze, Mariko Fitzgibbons, Bihong T. Chen

**Affiliations:** ^1^ Department of Radiology, Division of Neuroradiology City of Hope Comprehensive Cancer Center Duarte California USA; ^2^ Department of Radiology, Division of Neuroradiology Keck School of Medicine, University of Southern California Los Angeles California USA; ^3^ Department of Medical Oncology and Therapeutics Research City of Hope Comprehensive Cancer Center Duarte California USA; ^4^ Department of Pathology City of Hope Comprehensive Cancer Center Duarte California USA

**Keywords:** brain metastasis, breast cancer, dynamic contrast‐enhanced (DCE) perfusion, magnetic resonance imaging, volume transfer constant (K^trans^)

## Abstract

**Background:**

With the development of new human epidermal growth factor 2 (HER2)‐targeting therapies, a non‐invasive method of determining the HER2 status of breast cancer brain metastases can be of great clinical value, particularly given the risks of brain biopsy and the possibility of discordance between HER2 status of the primary breast cancer and the brain metastasis.

**Aims:**

The purpose of this study was to assess whether the volume transfer constant (Ktrans) from dynamic contrast‐enhanced (DCE) perfusion brain MR could assist in identifying the HER2 status of breast cancer brain metastases.

**Methods:**

With IRB approval for this retrospective study, we searched the electronic medical record at the City of Hope Comprehensive Cancer Center to identify all histopathologically proven breast cancer brain metastases with both preoperative DCE perfusion brain MR and HER2 assessment of the resected/biopsied brain specimens at the City of Hope Comprehensive Cancer Center from 2011‐2022. Mann‐Whitney tests were used to compare the Ktrans of the breast cancer brain metastases.

**Results:**

There were 9 women in our study cohort with a mean age of 55 years. Our cohort was comprised of a total of 9 breast cancer brain metastases, 3 of which were HER2‐positive, 6 of which were HER2‐negative. The Ktrans of HER2‐positive breast cancer brain metastases was significantly greater than the Ktrans of HER2‐negative breast cancer brain metastases (0.09 min^−1^ vs 0.02 min^−1^, U = 18.00, *p* = 0.024).

**Conclusion:**

Ktrans may help to differentiate HER2‐positive from HER2‐negative breast cancer brain metastases, if validated in a large prospective, multi‐center trial.

## 1 Introduction

Breast cancer is the most commonly diagnosed cancer in women internationally, with an estimated 2.3 million women diagnosed with breast cancer in 2020 [1]. Breast cancer accounted for approximately a quarter of all cancer cases in women and approximately 16% of cancer‐related deaths in women [1]. In 2020, it was estimated that approximately 685 000 women died from breast cancer. The incidence of breast cancer is expected to grow worldwide, with projections anticipating over 3 million new diagnoses of breast cancer per year and over 1 million breast‐cancer‐related deaths by 2040 [1].

The human epidermal growth factor 2 (HER2) status of breast cancers holds both prognostic and therapeutic significance in the management of breast cancer. HER2 is a transmembrane receptor oncoprotein that is involved in signal transduction cascades, such as the phosphatidylinositol 3‐kinase (PI3K) and mitogen‐activated protein kinase (MAPK) signal transduction pathways [2]. It has been reported that approximately 20% of breast cancers overexpress the HER2 protein (HER2‐positive) [3]. HER2‐positive breast cancers are an aggressive subtype of breast cancer that is associated with an increased incidence of systemic metastases, including brain metastases [3, 4, 5]. Approximately 10%–30% of breast cancer patients are diagnosed with brain metastases over the course of their disease [6]. HER2‐positive breast cancer patients have an even greater risk of brain metastases, as it has been reported that approximately 40% of HER2‐positive metastatic breast cancer patients will develop brain metastases at some point during the course of their disease [3]. The development of HER2‐targeting therapies, however, has improved the prognosis of patients with HER2‐positive breast cancers [2, 3]. Trastuzumab deruxtecan and tucatinib (in combination with trastuzumab and capecitabine), in particular, have been shown to be effective in treating HER2‐positive breast cancer brain metastases [7, 8, 9].

Identifying the HER2 status of breast cancer brain metastases is important for prognostic reasons, but it also defines candidacy for HER2‐targeted therapies. However, a challenge in the management of breast cancer patients is the possibility of a switch in HER2 status over time and between the primary breast cancer and metastatic lesions. The current management paradigm presumes that the HER2 status of the brain metastasis is the same as the HER2 status of the primary breast neoplasm. However, this presumption is wrong in approximately 10%–15% of cases [10, 11, 12, 13, 14]. Currently, the only definitive way to determine the HER2 status of breast cancer brain metastases relies upon pathologic tissue sampling via brain biopsy or resection with subsequent immunohistochemical (IHC) and/or fluorescent in situ hybridization (FISH) analyses. Because of these challenges, a noninvasive method of determining the HER2 status of breast cancer brain metastases can be of great value and can help improve the management of patients with breast cancer brain metastases.

A variety of non‐invasive imaging approaches have been used by multiple groups to identify imaging biomarkers for the HER2 status of breast cancer brain metastases. Ulu et al. utilized diffusion‐weighted MR (magnetic resonance) imaging to compare HER2‐positive and HER2‐negative breast cancer brain metastases and found that the minimum apparent diffusion coefficient value was significantly greater for HER2‐positive lesions in comparison to HER2‐negative lesions [15]. Kyeong et al. and Laakmann et al. examined spatial locations within the brain and found that HER2‐positive breast cancer brain metastases were more commonly located in the cerebellum, occipital lobes, and temporal lobes [16, 17]. In a prior study, we found that HER2‐positive breast cancer brain metastases exhibit a greater degree of enhancement on conventional contrast‐enhanced MR imaging than HER2‐negative breast cancer brain metastases [18]. We also found that tumor contour and tumor composition on conventional contrast‐enhanced MR are associated with the HER2 status of breast cancer brain metastases [19]. HER2‐positive lesions are more likely to have an irregular contour than HER2‐negative lesions. HER2‐positive lesions are also more likely to have a solid lesion composition than HER2‐negative lesions. In a prior study, we showed that relative cerebral blood volume (rCBV) derived from dynamic susceptibility contrast‐enhanced (DSC) perfusion MR is associated with the HER2 status of breast cancer brain metastases [20]. The rCBV of HER2‐positive lesions is significantly greater than the rCBV of HER2‐negative lesions. We also found that uptake on ^18^F‐fluorodeoxyglucose (^18^F‐FDG) positron emission tomography (PET) is associated with the HER2 status of breast cancer brain metastases [21]. HER2‐positive lesions exhibit a greater degree of the FDG uptake in comparison to HER2‐negative lesions.

While we do not have a definite mechanistic explanation for why HER2‐positive breast cancer brain metastases exhibit a greater degree of enhancement and a greater relative cerebral blood volume than HER2‐negative breast cancer brain metastases, we suspect that this may be related to increased vascular permeability and leakage of contrast from the blood–brain barrier. Dynamic contrast‐enhanced (DCE) perfusion MR imaging quantifies dynamic features of contrast leakage from the blood–brain barrier [22, 23]. It is most commonly utilized to measure the volume transfer constant (K^trans^), which is a transfer constant for the rate at which contrast leaks from the vasculature across the vascular endothelium into the extravascular‐extracellular space [22]. Because K^trans^ depends upon both the leakiness and surface area of capillaries, K^trans^ is often utilized as a marker for the tumor microvascular permeability [22, 23]. DCE perfusion MR has been used as a tool to predict treatment response in glioblastoma. For example, Kickingereder et al. found that baseline K^trans^ prior to bevacizumab therapy was predictive of survival outcomes (both progression‐free survival and overall survival) in glioblastoma patients [24]. K^trans^ has also been shown to have value in differentiating low‐grade gliomas from high‐grade gliomas [25]. Because the overexpression of HER2 in primary breast cancers is associated with periductal neovascularity and increased intratumoral microvessel density [26, 27], we suspect that K^trans^ may be associated with the HER2 status of breast cancer brain metastases. No currently published studies have investigated K^trans^ as a method of distinguishing HER2‐positive breast cancer brain metastases from HER2‐negative breast cancer brain metastases. The objective of this proof‐of‐concept pilot study was to assess whether K^trans^ derived from DCE perfusion MR could help to distinguish HER2‐positive breast cancer brain metastases from HER2‐negative lesions.

## 2 Materials and Methods

### 2.1 Study Cohort

City of Hope Comprehensive Cancer Center Institutional Review Board approval was received for this retrospective study; informed consent was waived. Inclusion and exclusion criteria for this study were formulated to identify all patients with histopathologically proven breast cancer brain metastases who had a preoperative DCE perfusion MR of the brain at the City of Hope Comprehensive Cancer Center during the time period of 2011 to 2022. We first searched the City of Hope Comprehensive Cancer Center's picture archiving and communication system (PACS) to identify all patients who received a contrast‐enhanced brain MR examination from 2011 to 2022 with the terms “breast cancer” and “perfusion” in the imaging report. This search identified 49 patients. The acquired MR images were then assessed for these 49 patients. For 24 of the 49 patients, the brain MR examinations did not include DCE perfusion MR images; thus, these 24 patients were excluded. Of the remaining 25 patients, 16 patients were excluded for lacking histopathologic confirmation of the breast cancer brain metastasis (via biopsy or resection) and thus lacking HER2 IHC and FISH analyses of the brain metastases. This resulted in a final cohort of nine patients. All of the patients in the cohort were women, with a mean age of 55 years. The age of the patients ranged from 32 years to 81 years. Each patient in our study cohort had one histopathologically proven breast cancer brain metastasis. The cohort thus included nine breast cancer brain metastases, each with HER2 IHC and FISH analyses of the breast cancer brain metastasis. DCE perfusion MR imaging was obtained prior to treatment for all lesions in our study cohort.

### 2.2 Acquisition of Brain MR Images

All brain MR images in our study were acquired on Siemens 3‐T scanners (MAGNETOM Verio, Biograph mMR, Siemens Medical Solutions, Erlangen, Germany). The following parameters were used for the acquisition of the DCE perfusion MR imaging data: acquisition sequence, 3D magnetization preparation rapid acquisition gradient‐echo (3D‐MPRAGE); TR, 9.3 ms; TE, 4.3 ms; flip angle, 15°; field of view, 192 mm × 280 mm; acquisition matrix, 81 × 192; slice thickness, 5 mm; gap thickness, 0 mm; number of slices, 16; number of dynamic phases, 50; temporal resolution, 5 s; total acquisition time, 4 min, 10 s. At the fifth time point, a power injector was utilized to intravenously administer 0.1 mmol/kg of gabodenate dimeglumine (Multihance, Bracco, Milan, Italy) or gabobutrol (Gadavist, Bayer, Leverkusen, Germany) at a rate of 3.5 mL/s, followed by a 30 mL bolus of saline solution. Pre‐contrast T1‐weighted images were acquired at three different flip angles (2°, 5°, and 10°) for absolute quantification of T1 relaxation time [28, 29].

Manufacturer‐supplied pulse sequences were also used to obtain standard MR imaging data. Following DCE perfusion MR imaging data acquisition, standard volumetric axial post‐contrast T1‐weighted images (axial MPRAGE images) were obtained. The size of each lesion was assessed by determining the maximal diameter of each lesion on the axial post‐contrast T1‐weighted images.

### 2.3 K^trans^ Measurement

DCE perfusion MR data was processed using the syngo.via platform (Siemens Medical Solutions, Erlangen, Germany), which utilizes the two‐compartment pharmacokinetic model developed by Tofts and Kermode [30]. K^trans^ color maps were calculated. Four circular regions of interest (ROIs) measuring 30–50 mm^2^ were placed in the regions of highest K^trans^ within each lesion by a fellowship‐trained board‐certified neuroradiologist with 7 years of experience. A senior fellowship‐trained board‐certified neuroradiologist with 22 years of experience then reviewed the ROIs for appropriate placement. Both neuroradiologists were blinded to the HER2 status of the lesions. The maximum value of the four measurements was recorded as the K^trans^ value of a lesion. This method of measurement has been employed by multiple groups to assess K^trans^ and other perfusion parameters for tumors [20, 25, 31, 32, 33, 34, 35, 36].

### 2.4 HER2 Assessment

The HER2 status of the lesions in our study cohort was determined by immunohistochemistry (IHC) and fluorescent in situ hybridization (FISH) of the resected and biopsied brain tumor specimens. These tests were interpreted by fellowship‐trained breast pathologists and board‐certified cytogeneticists in accordance with 2018 American Society of Clinical Oncology (ASCO)/College of American Pathologists (CAP) practice guidelines [18, 19, 20, 21, 37]. In accordance with the ASCO/CAP guidelines, a HER2 IHC score of 3+ was classified as HER2 IHC positive, a HER2 IHC score of 2+ was classified as HER2 IHC equivocal, and a HER2 IHC score of 0 or 1+ was classified as HER2 IHC negative. With regard to the HER2 FISH analyses, a breast cancer brain metastasis was classified as HER2 positive if the HER2/CEP17 (chromosome enumeration probe 17) ratio was greater than or equal to 2.0 or the average HER2 copy number was greater than or equal to 6.0 signals/cell. The estrogen receptor (ER) status, progesterone receptor (PR) status, and tumor grade of the resected and biopsied breast cancer brain metastases were obtained from pathology reports in the electronic medical record. For the two of the lesions in our study cohort, the tumor grade was not stated in the pathology report for the resected/biopsied breast cancer brain metastasis. For these two cases, the tumor grade was obtained from the pathology report for the primary breast cancer.

### 2.5 Statistical Analyses

We compared the K^trans^ values of the HER2‐positive and HER2‐negative breast cancer brain metastases using Mann–Whitney tests. We used a Mann–Whitney test to compare the lesion size between the two groups. We used Chi‐squared tests to compare the intracranial location, tumor grade, ER status, and PR status between HER2‐positive and HER2‐negative lesions. The threshold for statistical significance was a *p* value less than 0.05. We performed receiver operating characteristic (ROC) analyses to assess the performance of K^trans^ in distinguishing HER2‐positive from HER2‐negative lesions. SPSS 27 was used to perform the statistical analyses. In addition, we analyzed the data while only including breast cancer brain metastases that were imaged with gabodenate dimeglumine on the same Siemens MAGNETOM Verio 3 T scanner to account for possible effects from scanner and contrast agent differences.

## 3 Results

### 3.1 Study Cohort

The characteristics of the breast cancer brain metastases in our study cohort are presented in Table 1. Our study cohort was comprised of 3 HER2‐positive lesions and 6 HER2‐negative lesions. The mean time from DCE perfusion MR imaging to surgical specimen acquisition (resection/biopsy) was 6 days. The mean size of the HER2‐positive and HER2‐negative lesions was 3.8 cm and 3.2 cm, respectively; this difference in size was not statistically significant (U = 13.50, *p* = 0.26). We did not find any significant differences in the ER status (*χ* ^2^ = 0.90, *p* = 0.34), PR status (*χ* ^2^ = 0.56, *p* = 0.45), tumor grade (*χ* ^2^ = 0.32, *p* = 0.57), intracranial location (*χ* ^2^ = 1.50, *p* = 0.83), or contrast agent (*χ* ^2^ = 0.56, *p* = 0.45) between the HER2‐positive and HER2‐negative lesions. For nearly all of the lesions (8 of 9), MR images were acquired on the Siemens MAGNETOM Verio 3 T scanner. The MR images for the lone other lesion were acquired on the Siemens Biograph mMR 3 T scanner.

**TABLE 1:** Characteristics of breast cancer brain metastases.

Characteristic	All lesions	HER2‐positive	HER2‐negative
Mean age (years)^a^	55 (32–81)	51.3 (51.1–51.5)	57 (32–81)
Method of specimen acquisition			
Resection	5 (56)	1 (33)	4 (67)
Biopsy	4 (44)	2 (67)	2 (33)
Lesion size (cm)^a^	3.4 (2.1–6.2)	3.8 (2.9–4.4)	3.2 (2.1–6.2)
Intracranial location			
Frontal	3 (33)	1 (33)	2 (33)
Temporal	1 (11)	0 (0)	1 (17)
Parietal	2 (22)	1 (33)	1 (17)
Occipital	1 (11)	0 (0)	1 (17)
Cerebellum	2 (22)	1 (33)	1 (17)
Contrast agent			
Gadobenate dimeglumine	8 (89)	3 (100)	5 (83)
Gadobutrol	1 (11)	0 (0)	1 (17)
HER2 status by immunohistochemistry			
Positive	3 (33)	3 (100)	0 (0)
Negative	4 (44)	0 (0)	4 (67)
Equivocal	2 (22)	0 (0)	2 (33)
HER2 status by fluorescent in situ hybridization			
Positive	3 (33)	3 (100)	0 (0)
Negative	6 (67)	0 (0)	6 (100)
Estrogen receptor status by immunohistochemistry			
Positive	5 (56)	1 (33)	4 (67)
Negative	4 (44)	2 (67)	2 (33)
Progesterone receptor status by immunohistochemistry			
Positive	1 (11)	0 (0)	1 (17)
Negative	8 (89)	3 (100)	5 (83)
Tumor grade			
Grade 2	2 (22)	1 (33)	1 (17)
Grade 3	7 (78)	2 (67)	5 (83)

*Note:* Unless otherwise stated, data are the number of lesions, with percentages in parentheses.

Abbreviation: HER2, human epidermal growth factor receptor 2.

^a^ Data in parentheses are the ranges.

### 3.2 K^trans^

**FIGURE 1:**
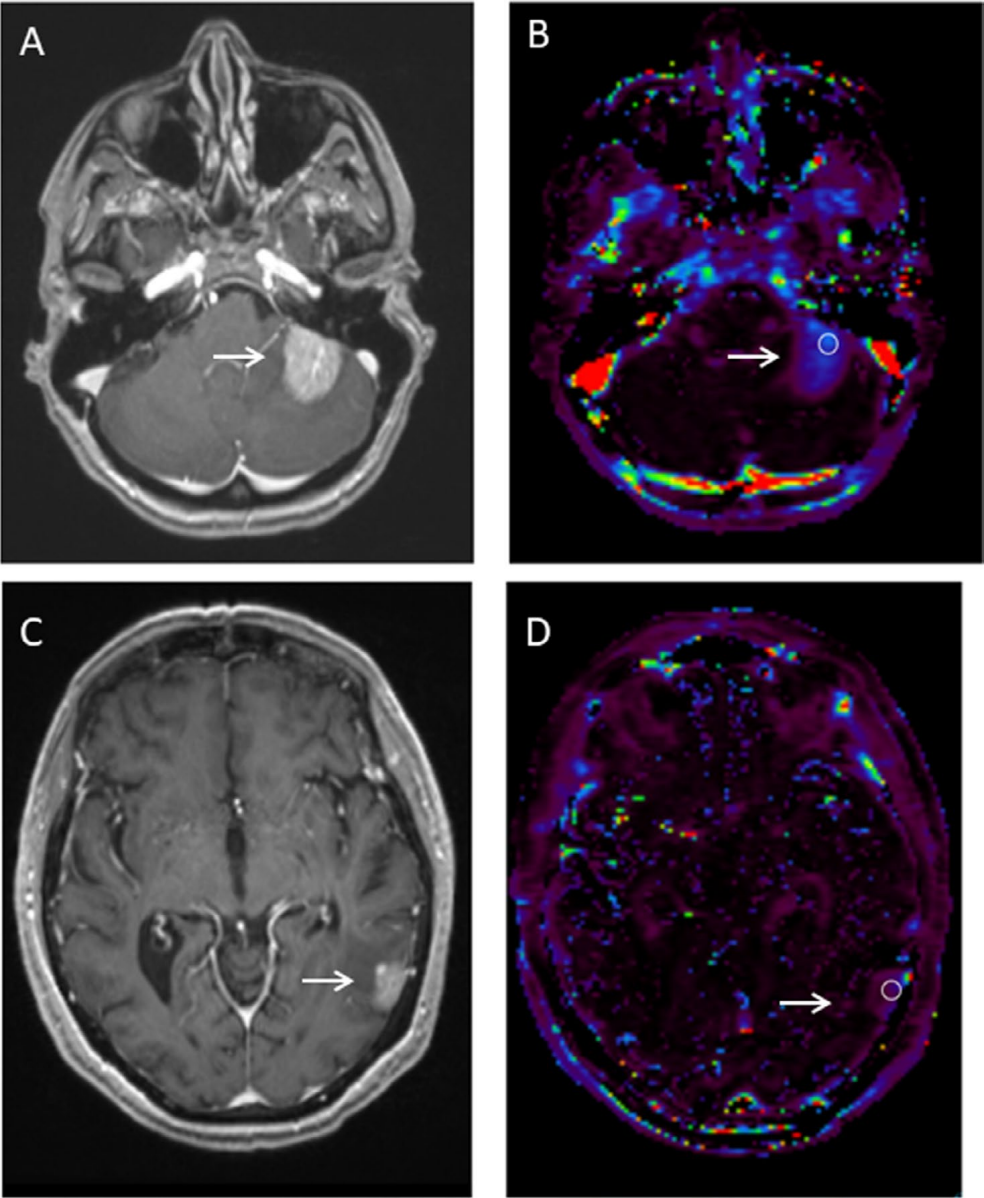
Axial T1‐weighted post‐contrast images and volume transfer constant (K^trans^) maps of HER2‐positive and HER2‐negative breast cancer brain metastases. Axial T1‐weighted post‐contrast image (A) and K^trans^ map (B) of a HER2‐positive breast cancer brain metastasis in a 51 year‐old woman (as shown by the black arrow). A representative region of interest on the K^trans^ map is shown in white. This breast cancer brain metastasis had a K^trans^ of 0.12 min^−1^. Axial T1‐weighted post‐contrast image (C) and K^trans^ map (D) of a HER2‐negative breast cancer brain metastasis in an 81 year‐old woman (as shown by the black arrow). A representative region of interest on the K^trans^ map is shown in white. This breast cancer brain metastasis had a K^trans^ of 0.03 min^−1^. HER2 = human epidermal growth factor receptor 2.

**FIGURE 2:**
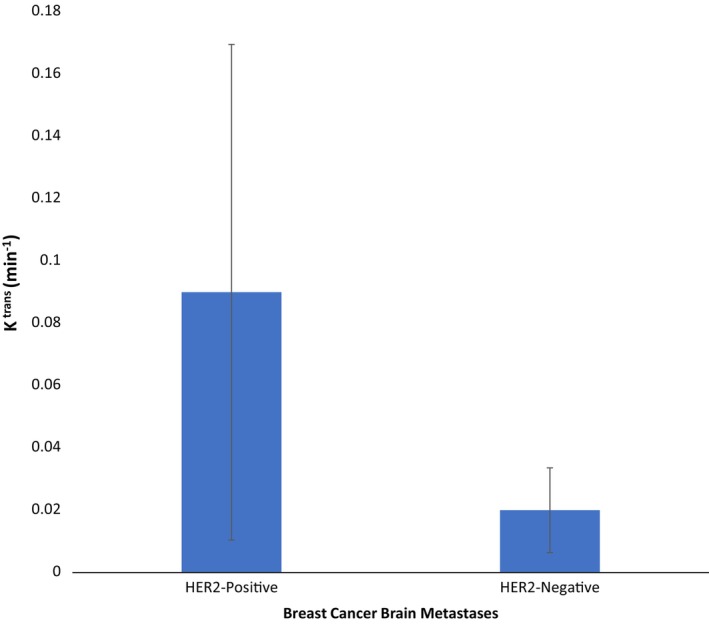
K^trans^ of HER2‐positive and HER2‐negative breast cancer brain metastases. Data points are K^trans^ values. Error bars are 95% confidence intervals. The K^trans^ of the HER2‐positive lesions was significantly greater than the K^trans^ of the HER2‐negative lesions (0.09 min^−1^ vs. 0.02 min^−1^, U = 18.00, *p* = 0.024). HER2 = human epidermal growth factor receptor 2.

As shown in Figures 1 and 2, the K^trans^ of the HER2‐positive lesions was significantly greater than the K^trans^ of the HER2‐negative lesions (0.09 min^−1^ vs. 0.02 min^−1^, U = 18.00, *p* = 0.024). ROC analyses showed that K^trans^ differentiated HER2‐positive from HER2‐negative lesions with an area under the curve of 1.00 (standard error < 0.001, *p* = 0.02). The accuracy‐maximizing threshold of 0.05 min^−1^ correctly classified all 9 lesions in our cohort.

We also analyzed the data while only including breast cancer brain metastases that were imaged with gabodenate dimeglumine on the Siemens MAGNETOM Verio 3 T scanner to account for possible effects from scanner and contrast agent differences. The results were similar. The K^trans^ of HER2‐positive lesions was significantly greater than the K^trans^ of HER2‐negative lesions (0.09 min^−1^ vs. 0.02 min^−1^, U = 15.00, *p* = 0.036).

## 4 Discussion

The goal of this study was to assess whether K^trans^ derived from DCE perfusion MR could help to distinguish HER2‐positive from HER2‐negative breast cancer brain metastases. We found that the K^trans^ of HER2‐positive lesions was significantly greater than the K^trans^ of HER2‐negative lesions. A K^trans^ threshold of 0.05 min^−1^ correctly classified all nine lesions in our study cohort. Our study is the first to report an association between K^trans^ derived from DCE perfusion MR and the HER2 status of breast cancer brain metastases.

The results of our study are consistent with our prior published study that found that HER2‐positive breast cancer brain metastases exhibit a greater degree of enhancement on conventional contrast‐enhanced MR than HER2‐negative lesions [18]. Our results are also consistent with our prior published study that found that HER2‐positive lesions have a greater rCBV derived from DSC perfusion MR than HER2‐negative lesions [20]. We suspect that the association between K^trans^ and HER2 status may be related to neovascularity‐associated vascular permeability. Claus et al. and Vameşu et al. found that the overexpression of HER2 in primary breast cancers is associated with periductal neovascularity and increased intratumoral microvessel density [26, 27].

There are several limitations to our study. First, this was a small exploratory, pilot study from a single institution assessing the association between K^trans^ and HER2 overexpression in breast cancer brain metastases. The reason for the small size of our study cohort was that we only included breast cancer brain metastases with histopathologic confirmation and with HER2 IHC and FISH analyses of the brain tumor specimens. A large number of the published studies evaluating imaging biomarkers of HER2 overexpression have correlated imaging features with the HER2 status of the primary breast neoplasm [15, 16, 17, 38], while our study correlated K^trans^ with the HER2 status of the resected/biopsied breast cancer brain metastases. This distinction is incredibly important because while many groups presume that the HER2 status of the brain metastasis is the same as the HER2 status of the primary breast neoplasm, this presumption is wrong in approximately 10%–15% of cases [10, 11, 12, 13, 14]. We understand that including only breast cancer brain metastases with histopathologic confirmation via brain biopsy or resection may not be entirely representative of the wider breast cancer population experiencing brain metastases. However, given the change in HER2 status from the primary breast neoplasm to the brain metastasis that occurs in approximately 10%–15% of cases, a biopsy or resection of the breast cancer brain metastasis is currently the only way to definitively establish the HER2 status of the breast cancer brain metastasis. Future studies should include prospective, multi‐center trials to validate our findings. Second, this was a retrospective study; thus, MR images for all of the lesions in our cohort were not acquired with the same contrast agent on the same MR scanner. Nevertheless, MR images for nearly all of the lesions in our cohort (eight of nine) were acquired with gadodenate dimeglumine on the Siemens MAGNETOM Verio 3 T scanner; MR images for the lone other lesion were acquired with gadobutrol on the Siemens Biograph mMR 3 T scanner. When we analyzed the data while only including breast cancer brain metastases that were imaged with gadobenate dimeglumine on the Siemens MAGNETOM Verio 3 T scanner, the results remained similar. Our results should be validated with a large prospective, multi‐center trial using a similar methodology to our retrospective proof‐of‐concept study; however, the MR data and the HER2 IHC and FISH analyses should be acquired prospectively at multiple centers with the same standardized MR protocol. Third, we did not have follow‐up DCE perfusion MR imaging for our cohort. Thus, we were unable to evaluate the longitudinal association between K^trans^ and HER2 overexpression in breast cancer brain metastases.

While HER2‐positive breast cancers have historically been associated with poorer outcomes, new HER2‐targeted therapies have been successful in improving outcomes for patients with HER2‐positive breast cancer. In particular, HER2‐directed therapies, such as trastuzumab deruxtecan and tucatinib, have been effective in treating brain metastases [7, 8, 9, 39]. Thus, these HER2‐targeted therapies can delay the need for other treatments with more prominent side effects, such as whole brain radiation and the associated cognitive impairment. Currently, the only definitive way to determine the HER2 status of breast cancer brain metastases relies upon pathologic tissue sampling to assess the HER2 status of breast cancer brain metastases. However, there are risks associated with brain tissue acquisition (via biopsy or resection). Given these circumstances, a non‐invasive method of identifying HER2 overexpression in breast cancer brain metastases is needed. K^trans^ derived from DCE perfusion MR may help to differentiate HER2‐positive from HER2‐negative breast cancer brain metastases if validated in a large prospective, multi‐center trial. K^trans^ may potentially provide a non‐invasive imaging complement to emerging liquid biopsy markers, such as cell‐free tumor DNA from cerebrospinal fluid (CSF) and CSF tumor cells, in the management of breast cancer brain metastases [40, 41].

## Author Contributions

**Jonathan R. Young:** conceptualization (lead), data curation (lead), formal analysis (lead), investigation (lead), methodology (lead), validation (lead), writing – original draft (lead), writing – review and editing (lead). **Luke N. Ledbetter:** conceptualization (supporting), data curation (supporting), investigation (supporting), methodology (supporting), writing – review and editing (supporting). **Julie A. Ressler:** conceptualization (supporting), data curation (supporting), investigation (supporting), methodology (supporting), writing – review and editing (supporting). **Mark S. Shiroishi:** conceptualization (supporting), investigation (supporting), methodology (supporting), writing – review and editing (supporting). **Joanne E. Mortimer:** conceptualization (supporting), investigation (supporting), methodology (supporting), writing – review and editing (supporting). **Daniel Schmolze:** conceptualization (supporting), data curation (supporting), investigation (supporting), methodology (supporting), writing – review and editing (supporting). **Mariko Fitzgibbons:** conceptualization (supporting), data curation (supporting), investigation (supporting), methodology (supporting), writing – review and editing (supporting). **Bihong T. Chen:** conceptualization (supporting), data curation (supporting), formal analysis (supporting), investigation (supporting), methodology (supporting), validation (supporting), writing – review and editing (supporting).

## Ethics Statement

City of Hope Comprehensive Cancer Center Institutional Review Board approval (20455) was obtained for this retrospective study; informed consent was waived. Consent for the de‐identified MR images in Figure 1 was waived in accordance with the guidelines of the National Institutes of Health (https://privacyruleandresearch.nih.gov/pr_08.asp#:~:text=How%20Can%20Covered%20Entities%20Use,for%20research%20on%20decedents'%20information).

## Conflicts of Interest

The authors declare no conflicts of interest.

## Data Availability

The data are available to qualified investigators upon reasonable request.
